# B Cell Development and T-Dependent Antibody Response Are Regulated by p38γ and p38δ

**DOI:** 10.3389/fcell.2020.00189

**Published:** 2020-03-24

**Authors:** Laura Barrio, Sara Román-García, Ester Díaz-Mora, Ana Risco, Rodrigo Jiménez-Saiz, Yolanda R. Carrasco, Ana Cuenda

**Affiliations:** Department of Immunology and Oncology, Centro Nacional de Biotecnología/CSIC, Madrid, Spain

**Keywords:** p38MAPK, p38γ, p38δ, B cell, lymphocyte, spleen

## Abstract

p38MAP kinase (MAPK) signal transduction pathways are important regulators of inflammation and the immune response; their involvement in immune cell development and function is still largely unknown. Here we analysed the role of the p38 MAPK isoforms p38γ and p38δ in B cell differentiation in bone marrow (BM) and spleen, using mice lacking p38γ and p38δ, or conditional knockout mice that lack both p38γ and p38δ specifically in the B cell compartment. We found that the B cell differentiation programme in the BM was not affected in p38γ/δ-deficient mice. Moreover, these mice had reduced numbers of peripheral B cells as well as altered marginal zone B cell differentiation in the spleen. Expression of co-stimulatory proteins and activation markers in p38γ/δ-deficient B cells are diminished in response to B cell receptor (BCR) and CD40 stimulation; p38γ and p38δ were necessary for B cell proliferation induced by BCR and CD40 but not by TLR4 signaling. Furthermore, p38γ/δ-null mice produced significantly lower antibody responses to T-dependent antigens. Our results identify unreported functions for p38γ and p38δ in B cells and in the T-dependent humoral response; and show that the combined activity of these kinases is needed for peripheral B cell differentiation and function.

## Introduction

B cell development from pluripotent haematopoietic stem cell to immature B cells in the bone marrow (BM), or from immature to mature B lymphocytes in the periphery, is regulated by multiple signalling pathways (Reth and Brummer, 2004). Failure of the appropriate signals occurring during B cell differentiation can lead to lymphomas or contribute to the development of autoimmune diseases (Jumaa et al., 2005). B cells are one of the major effectors of host defence against infections by producing antibodies that neutralise the invading pathogen. All these processes are mainly controlled by the stimulation of B-cell receptor (BCR), CD40 and/or chemokine and cytokine receptors. BCR and CD40 stimulation triggers the activation of signalling cascades, including the p38 mitogen-activated protein kinase (MAPK), which, in the case of BCR, has been shown to control B cell proliferation through the activation of the p38α substrate, the transcription factor MEF2C (Khiem et al., 2008; Wilker et al., 2008).

The mammalian p38MAPK family is composed of four members (p38α, p38β, p38γ, and p38δ) encoded by distinct genes; they are broadly expressed and activated by a wide range of cellular stresses and in response to inflammatory cytokines. These kinases share highly similar protein sequences and all are activated by phosphorylation mediated primarily by MAPK kinases (MKK)3 and MKK6 (Remy et al., 2010); they nonetheless differ in their expression patterns, substrate specificities and sensitivities to chemical inhibitors, and represent related, but clearly distinct p38MAPK subgroups (p38α/p38β and p38γ/p38δ) (Cuenda et al., 1997; Cuenda and Rousseau, 2007; Cuenda and Sanz-Ezquerro, 2017). p38α, the most abundant and best-characterised p38MAPK isoform, is activated during both innate and adaptive immune responses, and is a key regulator of the immune response (Gaestel et al., 2009; Rincon and Davis, 2009); evidence is, however, emerging that implicates p38γ and p38δ in this process (Cuenda and Sanz-Ezquerro, 2017).

p38γ and p38δ are expressed in immune cells (Hale et al., 1999; Fearns et al., 2000; Cuenda and Rousseau, 2007; Cuenda and Sanz-Ezquerro, 2017). Using mice deficient in p38γ, p38δ, or both, we and other have showed that these kinases play a crucial role in inflammation and in the immune response (Ittner et al., 2012; Risco et al., 2012, 2018; Cuenda and Sanz-Ezquerro, 2017; Alsina-Beauchamp et al., 2018). Both p38γ and p38δ modulate cytokine, chemokine and reactive oxygen species production; immune cell migration; or inflammasome and T cell activation (Ittner et al., 2012; Risco et al., 2012; Criado et al., 2014; Del Reino et al., 2014; Zur et al., 2015; Rajamaki et al., 2016; Cuenda and Sanz-Ezquerro, 2017; Alsina-Beauchamp et al., 2018). Recently we have found that the combined action of p38γ and p38δ is necessary for thymocyte development and peripheral T-cell homeostasis, indicating a role for these p38MAPK isoforms in the T-cell differentiation programme at the thymus (Risco et al., 2018). However, little is known about the implication of p38γ and p38δ in the normal physiology of other hematopoietic cells. No anomalies have thus far been reported in B cell development in p38γ-, p38δ-, or p38γ/δ-deficient mice. However, in a collagen induced arthritis (CIA) model, the reduced disease severity in p38γ/δ-deficient mice is associated with lower anti-collagen antibody production and responses than in control wild type mice, indicating that p38γ and p38δ regulate B-cell activation in CIA (Criado et al., 2014). Nonetheless, the role of p38γ and p38δ in B cell development and function remains unstudied.

In this work, we have performed a comparative analysis of the B cell differentiation in BM and spleen of WT mice, p38γ/p38δ-deficient mice and mice lacking p38γ and p38δ specifically in B cells. We also characterised in more detail the B cell activation in WT mice and mice lacking p38γ, p38δ or both. We found that the combined action of p38γ and p38δ is necessary for peripheral B cell development, BCR- and CD40-triggered B cell activation and proliferation, and for the ability of B cells to respond to T cell-dependent antigens.

## Materials and Methods

### Mice, Antibodies and Reagents

Mice lacking p38γ, p38δ and p38γ/δ have been described (Sabio et al., 2005; Risco et al., 2012). All strains were backcrossed onto the C57BL/6 strain for at least nine generations. B cell-specific p38γ/δ deficient (CD19-Cre^KI/+^p38γ/δ^f/f^) mice were generated by breeding p38γ/δ^f/f^ to CD19-Cre^KI/+^ mice (Rickert et al., 1997; Alsina-Beauchamp et al., 2018). Male and female 15–20 weeks old mice were used. Mice were housed in specific pathogen-free conditions in accordance with European Union regulations; work was approved by local CNB-CSIC ethical review and by the Bioethics Committee of the Community of Madrid PROEX316/15. *Escherichia coli* LPS was purchased from Sigma. Anti-p38α and TPL2 were from Santa Cruz. Antibodies to ERK1/2 and phospho-ERK1/2 (Thr202/Tyr204), Akt and phospho-Akt (Ser473, Thr408), phospho-NFκB1/p105 (Ser933; P-p105), phospho-p38MAPK (Thr180/Tyr182; this antibody recognises all four phosphorylated-p38 isoforms), IκBα and phospho-GSK3α/β (Ser21/9) were from Cell Signaling Technologies; anti-phospho-JNK1/2 (Thr183/Tyr185) was from Biosource. Anti-p38γ and -p38δ antibodies were raised and purified as described (Cuenda et al., 1997).

### Flow Cytometry Analysis

Thymus, spleen and lymph node cell suspensions were prepared; erythrocytes were lysed, and cells were counted. Cell samples were stained with combinations of fluorescently labelled antibodies to the cell surface markers CD19, CD3, CD4, CD8, CD43, CD25, Gr1, CD11b, B220, F4/80, IgD, CD21, CD23, CD69, CD86, CD95, GL7, PD-1 (all from BD Biosciences), CD138, CXCR5 (both from Biolegend) and IgM (Jackson Immunoresearch Lab.) for 30 min at 4°C. Cell analysis was performed in a FACScalibur, Beckman Coulter CYTOMIX FC500 MCL and LSR-II cytometer (BD Biosciences). Biotinylated goat anti-mouse IgG1, IgG2a, IgG2b, and IgG3 antibodies (Southern Biotech) were used to detect surface expression of IgG isotypes, followed by fluorescently labelled streptavidin (Molecular Probes). The profiles obtained were analysed with FlowJo (BD Biosciences) and Kaluza Analysis 2.11 (Beckman Coulter) software; B cells were gated as CD19^+^ cells when indicated.

### B Cell Isolation

Total splenocytes were obtained from freshly isolated spleens after tissue homogenisation and a Lympholyte step (Cedarlane Laboratories); residual erythrocytes were eliminated using erythrocyte lysis buffer (5 min, RT). For B cell isolation, total splenocytes were incubated with Dynabeads mouse pan-T (30 min, 4°C; Thy1.2, Dynal Biotech, Invitrogen) to eliminate the T cell fraction. The fraction enriched in B cells (>95% purity) was used for the *in vitro* experiments. Inguinal and popliteal lymph nodes were digested with collagenase-A plus DNAse-I (Roche; 15 min, 37°C), followed by homogenisation to isolate the lymphocyte compartment.

### B Cell Stimulation

Purified B cells were stimulated for various times with anti-BCR (1 μg/ml) or LPS (2.5 μg/ml). Cells were lysed in lysis buffer [50 μM Tris-HCl pH 7.5, 1 μM EGTA, 1 mM EDTA, 0.15 M NaCl, 1 mM sodium orthovanadate, 10 mM sodium fluoride, 50 mM sodium β-glycerophosphate, 5 mM pyrophosphate, 0.27 M sucrose, 0.1 mM phenylmethylsulphonyl fluoride, 1% (v/v) Triton X-100] plus 0.1% (v/v) 2-mercaptoethanol and complete proteinase inhibitor cocktail (Roche). Lysates were centrifuged (13,000 × g, 15 min, 4°C), supernatants removed, quick-frozen in liquid nitrogen, and stored at −80°C.

### Immunoblotting

Protein samples were resolved in SDS-PAGE and transferred to nitrocellulose membranes, which were blocked (30 min) in 50 mM Tris/HCl (pH 7.5), 0.15 M NaCl, 0.05% (v/v) Tween (TBST buffer) containing 10% (w/v) non-fat dry milk, then incubated in TBST buffer with 10% (w/v) non-fat dry milk and 0.5–1 μg/ml antibody (2 h, room temperature or overnight, 4°C). Protein was detected using horseradish peroxidase-conjugated secondary antibodies and the enhanced chemiluminescence reagent (Amersham Pharmacia Biotech), using the Odyssey infrared imaging system.

### Tissue Immunofluorescence

Freshly isolated spleens were immersed in OCT and frozen with liquid nitrogen. Cryostat sections (10 μm) were fixed in 4% PFA [10 min, room temperature (RT)], blocked with PBS containing 1% BSA and 10% goat serum (30 min, RT), and stained with FITC-conjugated rat anti-mouse IgD (BD Bioscience), Cy5-goat anti-mouse IgM (Jackson Immunoresearch) and biotin-rat anti-mouse CD169/MOMA-1 antibody (Acris Antibodies) plus Cy3-streptavidin (Jackson Immunoresearch), at the appropriate dilution in PBS 1% BSA (45 min, RT); washes were done with PBS. Sections were then mounted in Fluoromount (Southern Biotech) and imaged on a Zeiss Axiovert LSM 510-META inverted microscope with 10x/air objective. Images were analysed using LSM 510 software (Zeiss).

### Time-Lapse Microscopy on Planar Lipid Bilayers

Artificial planar lipid bilayers were prepared as previously described (Saez de Guinoa et al., 2011). In brief, unlabelled mouse GPI-linked ICAM-1-containing liposomes and/or liposomes containing biotinylated lipids were mixed with 1,2-dioleoyl-PC (DOPC) liposomes at various ratios to achieve specified molecular densities (ICAM-1 at 200 molecules/μm^2^; biotin-lipids, as indicated). Planar bilayers were assembled on sulphochromic solution-treated coverslips in FCS2 closed chambers (Bioptechs), then blocked with PBS 2% FCS (1 h, RT). For cell migration assays, ICAM-1-containing artificial bilayers were coated with recombinant murine CXCL13 (100 nM, 20 min, RT; Peprotech) immediately before imaging. For immune synapse formation assays, surrogate antigen (su-Ag) was anchored by incubating ICAM-1/biotin-lipids-containing membranes with AlexaFluor-647-streptavidin (Molecular Probes), followed by monobiotinylated rat anti-κ light chain mAb (BD Biosciences) (20 min, RT per incubation step). Monobiotinylation was obtained labelling the antibody with NHS-LC-LC-biotin at 1 μg/ml (30 min, RT, in PBS; Pierce), followed by dialysis and checked by flow cytometry using streptavidin-coated silica beads (5 μm-diameter; Bangs Laboratories). The number of GPI-ICAM-1 or anti-κ antibody molecules/μm^2^ was estimated using an immunofluorimetric assay and anti-ICAM-1 or anti-rat IgG antibodies, respectively. The standard values were obtained from microbeads with various calibrated rat IgG-binding capacities (Bangs Laboratories).

Violet-tracer-labelled (see below) WT B cells mixed with unlabelled p38γ/δ-deficient B cells at 1:1 ratio (2 × 10^6^), or the other way around, were injected into the warmed chamber (37°C) and imaged. Confocal fluorescence (1 μm-optical section), differential interference contrast (DIC) and interference reflection microscopy (IRM) images were acquired; to monitor cell motility, images were taken every 30 s for 20 min. Planar bilayers assembly at FCS2 chambers and assays with cells were performed in chamber-buffer (PBS 0.5%FCS, 0.5 g/l D-glucose, 2 mM MgCl_2_, 0.5 mM CaCl_2_). Images were acquired on an Axiovert LSM 510-META inverted microscope with a 40X oil immersion objective (Zeiss), at 512 × 512 pixels quality. Imaris 7.0 software (Bitplane) was used for qualitative and quantitative analysis of cell dynamics parameters, fluorescence and IRM signals. The fluorescence signal of the planar bilayer in each case was set up as the background of fluorescence intensity. The frequency of adhesion (IRM^+^ cells) per imaged field was estimated as [n° of B cells showing IRM contact/total n° of B cells (estimated by DIC)] × 100; similarly, we calculated the frequency of immune synapse (i.e., B cells showing IRM contact and forming a detectable su-Ag central cluster) or the frequency of migration (i.e., B cells showing an IRM contact and moving over time).

### *In vitro* Activation and Proliferation Assays

Total splenocytes or purified B cells (3 × 10^5^) were cultured in flat-bottom p96 wells alone or with anti-BCR [purified F(ab’)_2_ goat anti-mouse m heavy chain; Jackson Immunoresearch] at 1 or 10 μg/ml, hamster anti-mouse CD40 (clone HM40-3; BD Pharmingen) at 1 μg/ml or LPS (Sigma) at 2.5 μg/ml. After 24 h, cells were collected, stained for CD69, CD86, CD25, and CD19, and analysed by flow cytometry as described. For survival assays, purified B cells (3 × 10^5^) were cultured in flat-bottom p96 wells alone or with recombinant mouse BAFF (0.1 μg/ml; R&D Systems) and analysed for cell viability by flow cytometry after 24, 48, and 72 h; cell survival frequency was estimated by FSC/SSC morphological parameters. For proliferation assays, total splenocytes or purified B cells (5-10 × 10^6^ cells/ml) were labelled with 0.1 μM Violet Tracer (Molecular Probes) in PBS (10 min, 37°C); the labelling reaction was blocked with one volume of FCS (1 min, RT) and cells were washed with RPMI 10% FCS. Violet-labelled cells (3 × 10^5^) were cultured in flat-bottom p96 wells alone or with anti-BCR plus recombinant mouse IL-4 (20 ng/ml; Peprotech), anti-CD40 plus IL-4 or with LPS (Sigma) at the previously indicated doses. Cells were collected 96 h later, stained for CD19 for B cell identification, and analysed by flow cytometry. Assays were performed in RPMI 10% FCS. Proliferation index values were obtained from FlowJo analysis software (BD Biosciences); it is a ratio of the total number of divisions between the number of cells that went into division, both calculated at the time of analysis (96 h) in each experiment (see FlowJo website for more details).

For activation experiments with MZ and FO B cell subpopulations, spleens from WT and p38γδ^–/–^ mice were homogenised in PBS, treated with erythrocyte lysis buffer (5 min, RT), and then stained with FITC-conjugated rat anti-mouse CD21 and PE-conjugated rat anti-mouse CD23 (Biolegend; 30 min, 4°C). MZ (CD21^high^CD23^low^) and FO (CD21^+^CD23^+^) B cells were separated using a FACSAria Fusion flow cytometer and then cultured (5 × 10^4^) in round-bottom p96-wells plates in absence or presence of anti-BCR, anti-CD40 or LPS as described above. Cells were collected at 24 h, stained for CD69, CD86, and CD25, and analysed by flow cytometry.

### Chemotaxis Assays

Purified B cells (3 × 10^5^) from the spleen of WT or p38γ/δ-deficient mice were loaded on the top-reservoir of Boyden chambers (p24 transwell plates; 3 μm-diameter pore size; Costar) in 0.1 ml RPMI 10%FCS. In the bottom-reservoir, recombinant murine CXCL13 or CCL21 (10 μM; Peprotech) was added to 0.6 ml RPMI 10%FCS to get a final concentration of 400 and 100 nM, respectively. After 3 h in culture at 37°C, migratory B cells were collected from the bottom-well and counted by flow cytometry (1 min, at high flow rate). The migration frequency was estimated as [n° of migratory B cells at the bottom-well/n° of input B cells] × 100; the n° of input B cells was obtained by counting 3 × 10^5^ B cells in 0.6 ml of medium by flow cytometry. Assays were performed in duplicates.

### Immunisation

Groups of four WT mice or mice deficient in p38γ, p38δ or both were immunised i.p. with NP-KLH (50 μg; Biosearch Technologies) in alum (100 μl; Thermo Scientific) or with TNP-Ficoll (50 μg; Biosearch Technologies), in a final volume of 200 μl. Similarly, CD19-Cre^KI/+^ mice and B cell-specific p38γ/δ deficient (CD19-Cre^KI/+^p38γ/δ^f/f^) were immunised i.p. with NP-KLH in alum, as above. Peripheral blood samples were taken 1 day before immunisation, and at days 7, 14, 21, and 28 post-immunisation. Serum levels of NP- or TNP-specific antibodies of different isotypes (IgM, IgG1, IgG2a, IgG2b, IgG3, and IgA) were evaluated by ELISA. Briefly, ELISA plates were coated with NP-BSA (ratio > 20) or TNP-BSA (both from Biosearch Technologies), blocked with PBS 2% BSA, and incubated with appropriate dilutions of serum samples (1:1000 for IgM and distinct IgG). Bound antigen-specific antibodies were developed with biotin-goat anti-mouse Ig isotype-specific antibodies (Southern Biotech) plus alkaline phosphatase (AP)-streptavidin (Sigma); finally, we added the colorimetric substrate *p*-nitrophenyl phosphate (Sigma) and measured absorbance at 405 nm wavelength. To measure the affinity maturation of the anti-NP antibodies generated *in vivo*, ELISA plates were coated with NP3-BSA (low NP ratio) or with NP30-BSA (high NP ratio) (both from Biosearch Technologies) and followed by the same steps as above. The increase in antibody affinity was estimated by the ratio between the NP3-derived absorbance value and NP30-derived value in each case and at each time. Higher NP3/NP30-ratio values over time indicated increased antibody affinity (affinity maturation).

For the analysis of germinal centre (GC) and plasma cells (PC) populations, groups of five WT mice or mice deficient in p38γ/δ were immunised i.p. with NP-KLH in alum as explained above; spleens and bone marrow of these animals were analysed at day 7 post-immunisation by flow cytometry. The following antibody mixes against surface markers were used: B220/IgD/CD95/GL7/CXCR4/ef780 for GC B cells; B220/PNA/CD19/IgD/CD38/ef780 for GC and PC in spleen; B220/IgG1/CD138/IgD/Sca-1/IgM/ef780 for PC in spleen and BM; CD3/CD4/CXCR5/PD1 for follicular T helper cells. The ef780 viability dye (eFluor-780, Invitrogen) was used for dead cell exclusion in the analysis, and anti-CD16/32 for blocking non-specific staining via FCγ receptors.

### *In vitro* Activation Assays for Antibody Production

Purified B cells (1 × 10^5^) were cultured in round-bottom p96 wells alone or with anti-CD40 (1 mg/ml; clone HM40-3, BD Biosciences) plus IL-4 (20 ng/ml; Peprotech) or LPS (2.5 mg/ml; Sigma). After 96 h, supernatants were collected for antibody detection by ELISA; ELISA plates were coated with goat anti-κ light chain (1 μg/ml; Southern Biotech), blocked as before, incubated with appropriate dilutions of supernatants (1:100 for IgM; 1:10 for the distinct IgG), and developed as before.

### Statistical Analyses

Statistical data analysis between two groups was performed by applying the two-way ANOVA (immunisation analyses) and two-tailed unpaired Student’s *t*-test, using GraphPad Prism v. 7.0a. A *p*-value ≤ 0.05 was considered statistically significant.

## Results

### B Cell Development in p38γ- and p38δ-Deficient Mice

**FIGURE 1:**
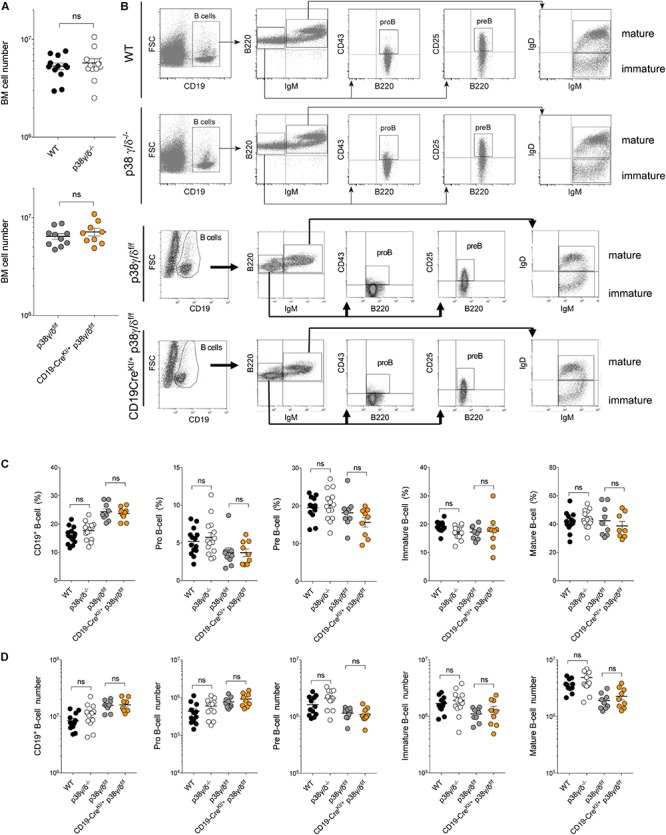
Analysis of B cell populations in BM from WT, p38γ/δ^–/–^, p38γ/δ^f/f^ and CD19-Cre^KI/+^-p38γ/δ^f/f^ mice. Cells were isolated from femoral and tibiae. **(A)** Total cell number in BM of the indicated genotypes. **(B)** Cell suspensions were stained with the indicated antibodies and analysed by flow cytometry. Representative flow cytometry profiles and analysis are shown. **(C)** Frequency and **(D)** total cell number of Pro-B cells (CD19^+^ IgM^–^ B220^+^ CD43^+^), Pre-B cells (CD19^+^ IgM^–^ B220^+^ CD25^+^), immature (CD19^+^ B220^+^ IgM^+^IgD^–^) and mature (CD19^+^ B220^+^ IgM^+^ IgD^+^) B cells in adult mouse BM. Percentage of each B-cell population was determined relative to CD19^+^ cell. Each dot in **(A,C,D)** represents a single mouse (*n* = 9–14). ns, not significant.

We examined the implication of p38γ and p38δ in B cell development comparing different B cell subpopulations by cytometry in the bone marrow (BM) of wild type (WT) mice, p38γ and p38δ deficient (p38γ/δ^–/–^) and B cell p38γ and p38δ deficient (CD19-Cre^KI/+^-p38γ/δ^f/f^). First, we checked, with specific antibodies, that WT B cells, but not B cells from CD19-Cre^KI/+^-p38γ/δ^f/f^ or p38γ/δ^–/–^ expressed p38γ and p38δ (Supplementary Figure S1A). We also confirmed that in CD19-Cre^KI/+^-p38γ/δ^f/f^ mice the expression of p38γ and p38δ was not affected in other tissues (Supplementary Figure S1B). The absolute cell numbers in BM were similar in p38γ/δ^–/–^ and WT mice, and in CD19-Cre^KI/+^-p38γ/δ^f/f^ and p38γ/δ^f/f^ mice (Figure 1A). p38γ/δ^–/–^ lymphocytes differentiated into pro-B, pre-B, immature and mature B cells in the BM (Figures 1B–D). All p38γ/δ^–/–^, CD19-Cre^KI/+^-p38γ/δ^f/f^, p38γ/δ^f/f^ and WT mice displayed similar frequency and absolute numbers of BM B cells (Figures 1C,D).

**FIGURE 2:**
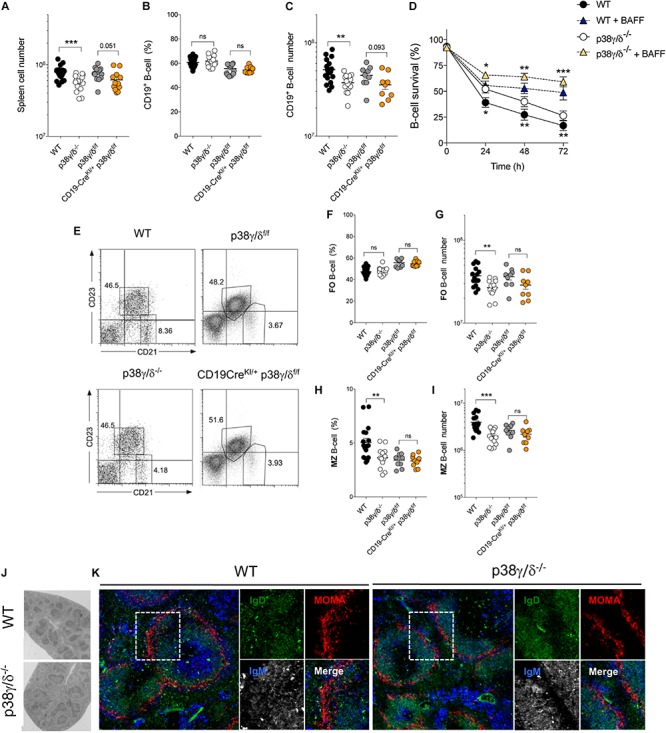
Analysis of B cell populations in the spleen. **(A)** Total cell number in spleens of adult mice of the indicated genotypes. Each dot represents a single mouse (*n* = 12–18). **(B)** B cell frequency and **(C)** total number in spleens of adult mice of the indicated genotypes; B cells were gated as CD19^+^ cells. Each dot represents a single mouse (*n* = 12–18). **(D)** Purified B cells were cultured in absence or presence of BAFF (0.1 μg/ml) and analysed for cell survival at the indicated time points by flow cytometry. B cell survival frequencies for each genotype are shown. Data are the mean ± SD of *n* = 3 mice per genotype. **(E)** Representative dot plots for CD21 and CD23 expression in splenocytes from mice of the specified genotypes. Frequencies of gated follicular (FO) B cells (CD21^+^CD23^+^) and marginal zone (MZ) B cells (CD21^hi^CD23^–^) (gates) are indicated. **(F,H)** Frequency and **(G,I)** total cell number of FO and MZ B cells in adult mouse spleens. Each dot represents a single mouse (*n* = 9-14). **(J)** Representative photomicrographs of hematoxylin/eosin-stained spleen sections from adult WT and p38γ/δ^–/–^ mice. **(K)** Fluorescence images of IgD (green), IgM (blue) and MOMA-1 (red) in representative tissue sections from spleens of WT and p38γ/δ^–/–^ mice. Right panels show a higher magnification of a B cell follicle and surrounding MZ. ns, not significant, *p ≤ 0.05, **p ≤ 0.01, ***p ≤ 0.001, *p*-value is also indicated in number.

We next evaluated the impact of p38γ and p38δ deletion on peripheral B cell development. In the spleen, p38γ/δ^–/–^ and CD19-Cre^KI/+^-p38γ/δ^f/f^ mice exhibited a decrease in total cell number compared to control mice (WT, p38γ/δ^f/f^ and CD19-Cre^KI/+^) (Figure 2A and Supplementary Figure S2A), that in the case of p38γ/δ^–/–^ was of ∼40% and statistically significant (Figure 2A). Accordingly, spleens from p38γ/δ^–/–^ mice were perceptibly smaller than those from WT mice (Supplementary Figure S2B). The deletion of either p38γ or p38δ alone did not affect spleen total cell number indicating a redundant role of these two kinases (Supplementary Figure S2C). Flow cytometry analysis of the B cell population showed no significant differences in B cell frequency (Figure 2B); however, p38γ/δ^–/–^ mouse spleens showed lower absolute numbers of B cells than those from WT mice (Figure 2C). The alterations in frequency and absolute B cell numbers were not significant in p38γ- or p38δ-deficient mouse spleens (Supplementary Figures S2D,E). The diminished B cell number in the spleen of p38γ/δ^–/–^ mice was not due to impaired B cell-activating factor (BAFF) signalling, since the response to BAFF survival signal of p38γ/δ deficient B cells and WT B cells was similar (Figure 2D). In addition, absolute numbers, but not frequency, of other splenic immune cell populations from p38γ/δ^–/–^ mice were perceptibly smaller than those from WT mice (Table 1 and Supplementary Figure S2F). This is consistent with the decrease in total spleen cell number and indicates that the reduced B cell number in p38γ/δ^–/–^ mouse spleens is due to a decreased size of the organ.

**TABLE 1:** Lymphoid and myeloid cell population in the spleen of p38γ and p38δ deficient mice.

	**WT**	**p38γ/δ^–/–^**
CD3^+^	37.60 ± 4.57 (*n* = 18)	35.98 ± 9.33 (*n* = 16; *p* = 0.48)
CD3^+^CD4^+^	22.29 ± 3.86 (*n* = 17)	19.69 ± 5.08 (*n* = 16; *p* = 0.08)
CD3^+^CD8^+^	13.02 ± 2.52 (*n* = 17)	12.20 ± 2.58 (*n* = 16; *p* = 0.32)
CD11b^+^	15.55 ± 9.50 (*n* = 20)	9.87 ± 3.80 (*n* = 16; *p* = 0.02)
CD11b^+^F4/80^+^	8.70 ± 7.14 (*n* = 20)	5.13 ± 3.65 (*n* = 16; *p* = 0.06)
CD11b^+^Gr1^+^	8.14 ± 6.06 (*n* = 20)	3.71 ± 1.84 (*n* = 16; *p* = 0.004)

*Spleen cells form WT and p38**γ**/**δ**^–/–^ mice were stained with anti-CD3, -CD4, -CD8, -CD11b, -Gr1 and -F4/80. The percentage of the indicated cell population was examined by flow cytometry. *n* is the number of mouse spleen analysed; *p*-values are referred to WT.*

Conventional mature B cells are classified into two subsets in the mouse, follicular (FO) and marginal zone (MZ). Recirculating FO B cells are usually found in lymphoid follicles of the spleen and lymph nodes, while MZ B cells reside primarily around the periphery of spleen lymphoid nodules. Using cell staining and flow cytometry techniques, we analysed the two main spleen B cell subpopulations separately, FO B cells (CD21^+^CD23^+^) and MZ B cells (CD21^high^CD23^–^) (Figure 2E). FO B cell frequency was not markedly affected by the absence of p38γ and p38δ in either the whole mouse or specifically in B cells (Figure 2F), whereas absolute numbers were lower in p38γ/δ^–/–^ mice as expected (Figure 2G). Both, MZ B cell frequency and absolute numbers were markedly reduced in p38γ/δ^–/–^ spleens compare to WT (Figures 2H,I); however, there were not differences between MZ B cell frequency and absolute numbers from CD19^KI/+^-p38γ/δ^f/f^ and p38γ/δ^f/f^ mice (Figures 2H,I). The lower MZ B cell frequency observed in p38γ/δ^f/f^ mice compared to WT mice could be due to the reduced levels of p38γ and p38δ protein in those mice (Supplementary Figure S1). p38γ^–/–^ and p38δ^–/–^ mice also had lower MZ B cell frequencies and total cell number than WT mice, although the differences were not statistically significant (Supplementary Figures S2G,H). FO B cell frequency and absolute cell number were similar in p38γ^–/–^, p38δ^–/–^ and WT mice (Supplementary Figures S2G,H). The MZ B cell compartment in p38γ/δ^–/–^ mice was almost half the size found in WT counterparts (Figure 2I), which implicated p38γ and p38δ in MZ B cell differentiation in spleen.

MZ B cells are located around the follicles in the spleen. To examine the white pulp structure and MZ B cell localisation, *in situ*, we performed H&E and immunofluorescence staining in cryostat sections of spleens from p38γ/δ^–/–^ and WT mice. Spleen architecture was maintained in p38γ/δ^–/–^ compared to WT mice, and white and red pulps were similar (Figure 2J). Staining with anti-IgM and -IgD revealed a normal B cell distribution in the white pulps of p38γ/δ^–/–^ spleen (Figure 2K). To visualise the MZ, we stained with anti-MOMA-1, which stains the metallophilic macrophages located at the boundary between the FO and the MZ. Despite the B cell deficiencies in p38γ/δ^–/–^ mice, we detected no alterations in the formation of B cell follicles (IgM^+^IgD^+^) or MZ B cell (IgM^+^IgD^–^) location near MOMA-1^+^ metallophilic macrophages (Figure 2K).

### Migration and Immune Synapse Formation of p38γ/p38δ Deficient B Cells

**FIGURE 3:**
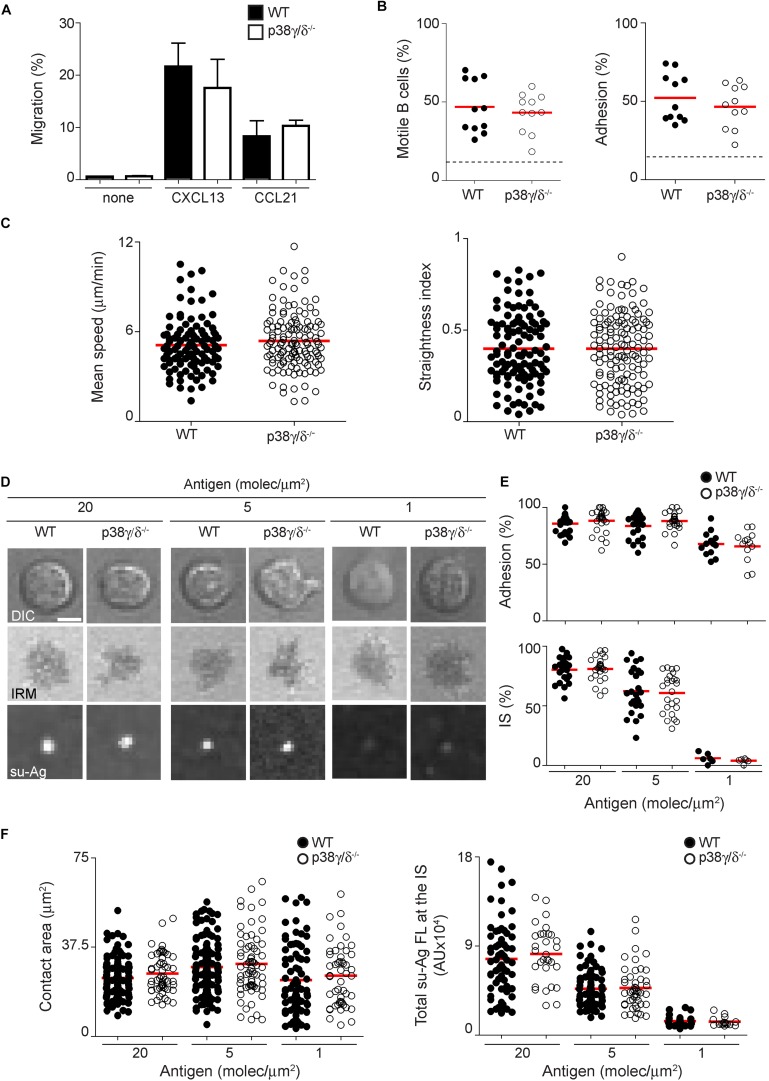
Migration and immune synapse formation of p38γ/δ deficient B cells. **(A)** B cells were cultured in absence (none) or presence of CXCL13 or CCL21 gradients, in Boyden chambers, during 3 h. Migratory B cells were collected and counted by flow cytometry. Migration frequencies for each condition and genotype are shown; data are the mean ± SD of *n* = 3 mice per genotype. **(B,C)** WT and p38γ/δ deficient B cells were settled on ICAM-1-containing planar lipid bilayers uncoated or CXCL13-coated, and monitored for cell adhesion and motility by real-time microscopy. **(B)** Frequencies of B cell migration (left panel) and adhesion (right panel); data are from two independent experiments (*n* = 4 mice per genotype). Dashed line indicates mean value of basal migration/adhesion (in absence of CXCL13). **(C)** Values of mean speed (left panel) and straightness index (right panel) for motile B cells; data from representative experiment are shown (*n* = 4 mice per genotype). **(D–F)** WT and p38γ/δ deficient B cells were settled on ICAM-1-containing planar lipid bilayers in the presence of tethered su-Ag at the specified densities, and monitored for IS formation. **(D)** DIC, IRM and fluorescence su-Ag images at the contact plane of representative IS-forming B cells. Bar, 2 μm. Data acquisition was performed at medium quality (512 × 512 pixels). **(E)** Frequencies of B cell adhesion (upper panel) and su-Ag central cluster (cSMAC; bottom panel). **(F)** Values of contact area (IS area; in μm^2^; left) and total su-Ag fluorescence at the IS (in arbitrary units, AU; right) for B cells with established IS in each case. Data from a representative experiment are shown in **(E,F)** (*n* = 3 mice per genotype). Each dot in **(B,E)** represents a single image field, and in **(C,F)** a single cell.

Chemokine-triggered B cell migration is essential for the B cell response. It facilitates homing to secondary lymphoid organs and scanning of the follicular stromal cell network where B cells find the antigen. To evaluate if the diminished B cell numbers at spleen of p38γ/δ^–/–^ mice might be related with impaired migration and homing, we studied B cell motility in response to chemokine stimulation by two different approaches. We analysed the effect of the lack of p38γ and p38δ on the B cell response to CXCL13 or CCL21 chemokine gradients using Boyden chambers (see methods); both chemokines are involved in B cell migration *in vivo*. The chemotactic response of p38γ/δ^–/–^ B cells was equivalent to that of WT for both chemokines (Figure 3A). We next addressed adhesion-dependent B cell motility using a two-dimensional model based on artificial planar lipid bilayers, combined with real-time interference reflection microscopy (IRM) and confocal fluorescence microscopy; this model mimics *in vivo* B cell motility within the follicles (Saez de Guinoa et al., 2011). We isolated WT and p38γ/δ^–/–^ B cells and assayed their migratory capacity on CXCL13-coated, intracellular adhesion molecule (ICAM)-1-containing membranes. p38γ/p38δ deficiency did not seem to cause any impairment in either B cell migration or adhesion frequency (Figure 3B); moreover, both mean speed and directionality (measured as straightness index) were similar in p38γ/δ^–/–^ and WT splenic cells (Figure 3C).

BCR recognition of antigen leads to immune synapse (IS) formation at the B cell/antigen-presenting cell (APC) interface. The IS framework supports signalling and polarised membrane trafficking to reach B cell activation and antigen extraction from the APC. We evaluated the role of p38γ and p38δ on B cell IS formation by tethering su-Ag (anti-κ light chain antibody; su-Ag) to ICAM-1-containing artificial planar lipid bilayers. We monitored IS formation in the presence of different su-Ag density using IRM and confocal fluorescence microscopy, to measure the B cell contact with the artificial bilayer (adhesion; IS area) and su-Ag central clustering, respectively (Figure 3D). At 20 molecules/μm^2^ su-Ag density, the frequency of B-cell adhesion and su-Ag clustering is almost 100%, whereas at 1 molecules/μm^2^ su-Ag density, B-cell adhesion was approximately 60% and su-Ag central cluster was hardly detected (Figures 3D,E). These results were similar with those previously described (Saez de Guinoa et al., 2011). The frequency values of p38γ/δ^–/–^ B cells were equivalent to those for WT B cells (Figures 3D,E). Additionally, either IS contact areas with the planar bilayer, detected by IRM, or total su-Ag aggregation at the IS were not affected by the lack of p38γ/p38δ (Figure 3F). Overall, our data indicated that p38γ and p38δ are not involved in CXCL13/CCL21-triggered B cell migration or BCR-promoted IS formation.

### Role of p38γ and p38δ Isoforms in BCR- TLR4- and CD40-Triggered B Cell Activation

**FIGURE 4:**
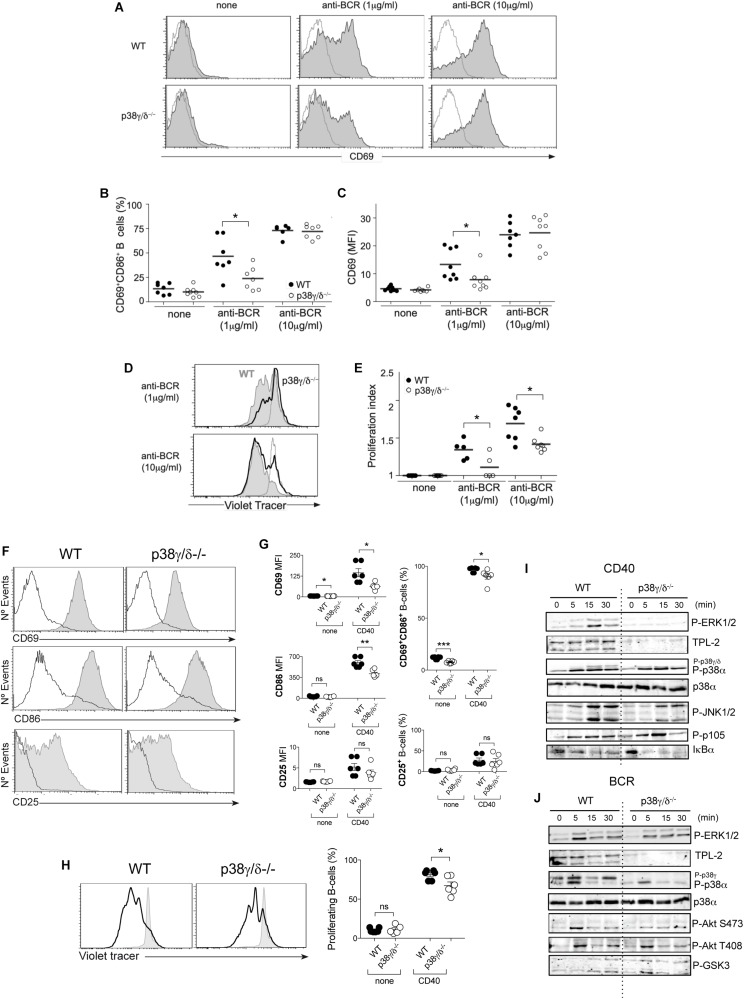
B cell activation in the absence of p38γ and p38δ. **(A–C)** Splenocytes from WT (black) or p38γ/δ^–/–^ (white) mice were cultured alone (none) or with anti-BCR (1 and 10 μg/ml) for 20 h. CD19^+^-gated B cells were analysed for expression of CD69 and CD86 activation markers by flow cytometry. **(A)** Representative profiles of surface CD69 expression (filled histograms) in B cells stimulated with the indicated ligands; grey line, isotype control. **(B)** Frequency of activated B cells, gated as CD69^+^CD86^+^, in the total B cell population for each condition. **(C)** CD69 mean fluorescence intensity (MFI) values in CD19^+^-gated B cells. In **(B,C)** each dot represents a single mouse (*n* = 7–8). **(D,E)** Violet tracer-labelled splenocytes of the specified mouse genotypes were cultured alone (none) or with anti-BCR (1 and 10 μg/ml) plus IL-4 (20 ng/ml) for 96 h. CD19^+^-gated B cells were analysed for violet tracer signal dilution by flow cytometry. **(D)** Representative violet tracer profiles of WT (filled histograms) and p38 isoform-deficient (black line) B cells stimulated as above; grey line, violet tracer profile of unstimulated B cells after 96 h in culture. **(E)** Proliferation index of B cells in each condition; values obtained with FlowJo software. Each dot represents a single mouse (*n* = 4-7). **(F,G)** Splenocytes from WT or p38γ/δ^–/–^ (white) mice were cultured alone or in presence of anti-CD40 (1 μg/ml) for 20 h. **(F)** Representative profiles of surface CD69, CD86, and CD25 expression (filled histograms) in CD40-stimulated B cells. Grey line indicates unstimulated B cells at 20 h. **(G)** Frequency of CD69^+^CD86^+^ B cells (top, right) and of CD25^+^ B cells (bottom, right), and MFI values of CD69, CD86 and CD25 (left panels) for CD19^+^-gated B cells in each condition (none, unstimulated; CD40, anti-CD40). **(H)** Splenocytes were cultured alone (none) or in presence of anti-CD40 (1 μg/ml) plus IL-4 (20 ng/ml) for 96 h. Left, representative violet tracer profiles of WT and p38γ/δ deficient B cells (black line); filled histogram, unstimulated B cells at 96 h. Right, frequencies of proliferating B cells (with diluted violet tracer level) in each condition. Each dot in **(G,H)** represents a single mouse (*n* = 5-6). **(I,J)** Purified B cells from WT or p38γ/δ^–/–^ mice were unstimulated or stimulated with **(I)** anti-CD40 (1 μg/ml) or **(J)** anti-BCR (1 μg/ml) for the times indicated and cell lysates immunoblotted with the indicated antibodies using the Odyssey infrared imaging system. Figure shows representative immunoblots from three independent experiments. ns, not significant, *p < 0.05, **p ≤ 0.01, ***p ≤ 0.001.

Since p38γ and p38δ have a role in the regulation of immune cell signalling and gene expression (Cuenda and Sanz-Ezquerro, 2017) we analysed the responsiveness of B cells from WT and p38γ/δ^–/–^ mice to both T-independent and -dependent stimuli. B cell activation by anti-BCR, LPS or anti-CD40 leads to upregulation of several cell surface proteins, including CD69 and the costimulatory molecule CD86. We performed BCR and TLR4 stimulation experiments with total splenocytes or purified B cells, and obtained similar B cell activation and proliferative responses (data not shown); for practical reasons, the following assays were done using splenocytes, and B cells were gated as CD19^+^ cells. To determine the effect of p38γ and p38δ deficiency on CD69 and CD86 expression, we cultured splenocytes from WT or p38γ/δ^–/–^ mice alone or with different doses of soluble su-Ag [F(ab’)_2_ anti-μ heavy chain antibodies] for B cells; 24 h later, we analysed B cells for surface expression of the activation markers. At a 1 μg/ml su-Ag dose, p38γ/δ^–/–^ B cell activation was reduced compared to WT B cells (Figures 4A–C); p38γ^–/–^ B cells also had BCR-triggering defects at this antigen dose, suggesting a prominent role for p38γ than for p38δ downstream of BCR signalling (Supplementary Figures S3A–C). We nonetheless observed no defects in BCR-triggered B cell activation at a higher su-Ag dose (10 μg/ml; Figures 4A–C). The results indicate that, above a BCR signalling threshold, B cells counteract the effect of p38γ and p38δ deficiency in early B cell activation events (CD69 and CD86 upregulation).

We then evaluated later events in B cell activation using *in vitro* proliferation assays. Violet tracer-labelled splenocytes were stimulated with two doses of su-Ag in the presence of IL-4, and analysed by flow cytometry 72 h later for violet tracer signal. CD19^+^ gated B cells from p38γ/δ^–/–^ mice proliferated significantly less than those from WT mice, independently of the antigen dose (Figures 4D,E). Neither p38γ^–/–^ nor p38δ^–/–^ B cells showed defects in BCR-triggered proliferation rates (Supplementary Figure S3D). These data indicated that both p38γ and p38δ participate in the BCR signalling cascade that drives B cell activation and proliferation. Whereas p38γ appears to be more important in early activation events (Supplementary Figures S3A–C), each isoform compensates for the other in antigen-mediated B cell proliferation; only the absence of both p38γ and p38δ decreased proliferation in response to su-Ag.

In *in vitro* activation assays using the TLR4 ligand LPS as a stimulus, we did not detect any significant variation in p38γ/δ^–/–^ B cell activation frequency (CD86^+^CD69^+^) compared to WT; CD69 levels were, however, reduced (Supplementary Figures S3E,F). Lack of p38γ alone also affected CD69 upregulation in LPS stimulated B cell activation, indicating the involvement of this isoform in LPS-induced B cell activation. To evaluate the need for p38γ and p38δ in TLR4-induced proliferation, we stimulated splenocytes with LPS. LPS-triggered CD19^+^-gated B cell proliferation was similar in p38γ/δ^–/–^, p38γ^–/–^, p38δ^–/–^ and WT B cells (Supplementary Figure S3G), indicating that in these conditions, p38γ and p38δ do not have a central role in the TLR4 signalling cascade that drives B cell proliferation.

CD40 activation of B cells causes the expression of CD25 as well as high levels of CD69 and the co-stimulatory molecule CD86. We did not find any significant variation in the expression of CD25 in p38γ/δ^–/–^ B cell compared to WT after CD40 stimulation (Figures 4F,G); CD69 and CD86 levels and CD86^+^CD69^+^ frequency were however reduced (Figures 4F,G), indicating the involvement of these p38MAPK isoforms in CD40-induced B cell activation. CD40-mediated proliferation was slightly, but significantly, reduced in p38γ/δ^–/–^ B cells (Figure 4H), suggesting that in these conditions, p38γ and p38δ regulate the CD40 signalling cascade that drives B cell proliferation.

Since the MZ B cell compartment in p38γ/δ^–/–^ spleens is reduced compared to WT (Figures 2H,I), we examined if the activation of either MZ B cells or FO B cells was affected by the lack of p38γ and p38δ. We flow-sorted MZ and FO B cells from both WT and p38γ/δ^–/–^ mice (Supplementary Figure S4A), and performed comparative analysis of CD69, CD86 and CD25 expression in response to different stimuli (anti-BCR, anti-CD40 and LPS) (Supplementary Figures S4B,C). We found that CD69, but not CD86 expression was decreased in both p38γ/δ^–/–^ MZ and FO B cells compared to WT MZ and FO B cells, whereas CD25 expression in response to anti-CD40 stimulation was not affected (Supplementary Figures S4B,C). These results indicate that p38γ and p38δ regulate the activation of both MZ B and FO B cells.

p38γ and p38δ regulate TLRs and Dectin-1 signalling in macrophages by impairing ERK1/2 pathway activation (Risco et al., 2012; Alsina-Beauchamp et al., 2018), which is central for cellular proliferation; we thus examined BCR and CD40 signalling using phosphospecific antibodies to detect activated ERK1/2 and also p38MAPK by immunoblot. Both BCR and CD40 stimulation led to ERK1/2 activation in WT cells, and lack of p38γ and p38δ impaired the activation of ERK1/2 induced by CD40, but not by anti-BCR (Figures 4I,J). CD40 activation of ERK1/2 is mediated by the upstream MEK kinase, TPL2 (Eliopoulos et al., 2003; Papoutsopoulou et al., 2006); p38γ/p38δ deletion decrease TPL2 protein levels in B cells (Figure 4I), therefore, it is possible that the impaired ERK1/2 activation in p38γ/δ^–/–^ B cell is due to the lack of the upstream kinase TPL2, similar to what happen in TLR- and Dectin-1-mediated ERK1/2 activation (Risco et al., 2012; Alsina-Beauchamp et al., 2018). In contrast, CD40-induced p38α, JNK and IKK pathways activation were not affected in p38γ/δ^–/–^ B cell (Figure 4I). This finding suggests a role for ERK1/2 pathway in the decreased proliferation in p38γ/p38δ-deficient B cells in response to CD40, not in response to BCR activation. Since Akt pathway is also implicated in B-cell proliferation and it is one of the main pathways implicated in BCR signalling (Rickert, 2013), we measured Akt activation in response to BCR, and found that the phosphorylation of Akt and its downstream target GSK3 were similar in WT and p38γ/δ^–/–^ B cell (Figure 4J). p38α phosphorylation was not impaired in p38γ/δ^–/–^ B cell in response to BCR activation (Figure 4J). These results suggest that, although p38γ and p38δ do not control the activation of ERK1/2 and Akt pathways in response to BCR, they could be directly regulating the activity of other downstream protein essential for B cell proliferation.

### Role of p38γ and p38δ Isoforms in the B Cell Antibody Response *in vivo*

**FIGURE 5:**
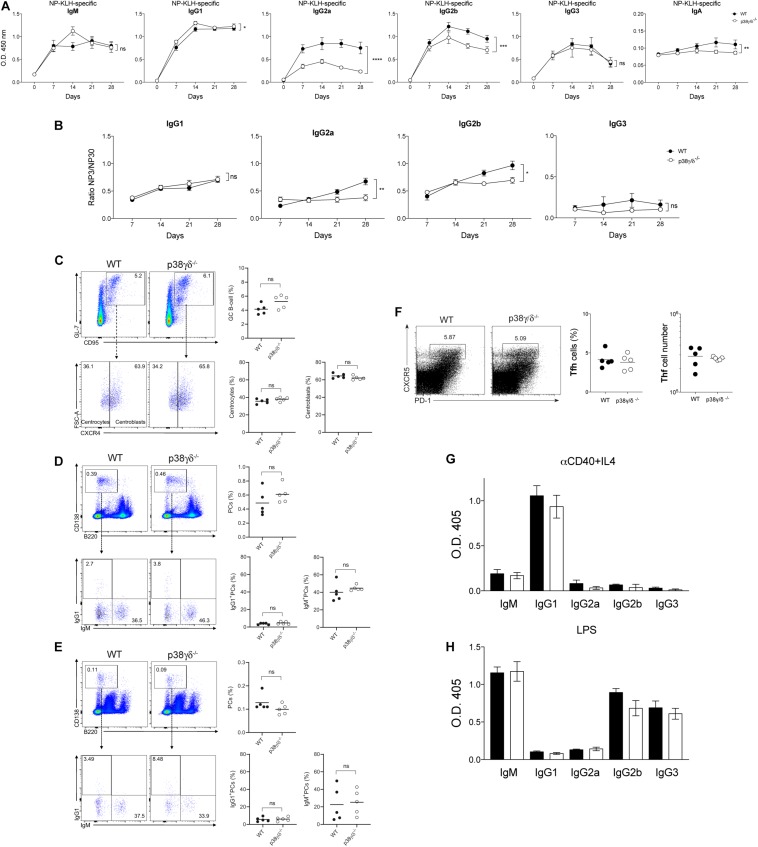
Antibody response to T-dependent antigen in p38γ- and p38δ-deficient mice. **(A)** Adult WT and p38γ/δ^–/–^ mice were immunised i.p. with NP-KLH in alum (T-dependent). Serum samples were analysed by ELISA for TNP-specific antibodies of different isotypes (IgM, IgG1, IgG2a, IgG2b, IgG3, IgA) at the indicated times. O.D., optical density. **(B)** Serum samples from NP-KLH immunised mice were analysed for the presence of NP-specific high affinity antibodies, using NP_3_ as substrate compared to NP_30_. Values of the ratio between NP_3_-ELISA and NP_30_-ELISA for the indicated IgG isotypes in each time point and for each mouse are shown. Each dot in **(A,B)**, represents a mouse (*n* = 4 mice/group); ns, not significant, *p ≤ 0.05, **p ≤ 0.01, ***p ≤ 0.001, ****p ≤ 0.0001. **(C–F)** Adult WT and p38γ/δ^–/–^ mice were immunised i.p. with NP-KLH in alum. Spleens and BMs were analysed at day seven post-immunisation for the presence of GC B cells (B220^+^ IgD^neg/low^ GL7^+^ CD95^+^; CXCR4^high^ for centroblast and CXCR4^+/low^ for centrocytes), plasma cells (PC; CD138^high^ B220^low^, IgM^+^ or IgG1^+^), and T follicular helper cells (Tfh; CD3^+^ CD4^+^ CXCR5^+^ PD1^+^) by flow cytometry (gating strategy shown in Supplementary Figures S6A,C). Representative dot plots of **(C)** GL7/CD95 for B220^+^ IgD^neg/low^-gated B cells (top panels), and FSC-A/CXCR4 for GL7^+^ CD95^+^-gated B cells (bottom panels); **(D)** CD138/B220 for total splenocytes (top panels), and IgG1/IgM for CD138^high^ B220^low^-gated PC (bottom panels); **(E)** as in **(D)** but for total BM; and **(F)** CXCR5/PD-1 for CD3^+^CD4^+^-gated T cells, are shown. Regions used for GC, PC and Tfh analysis are indicated in each case. Frequencies of the analysed populations are shown (right panels); each dot is a mouse. Data are from one experiment (*n* = 5 mice per group). **(G,H)** B cells isolated from spleens of WT and p38γ/δ^–/–^ mice were cultured with **(G)** anti-CD40 mAb plus IL-4 (T-dependent stimulation) or **(H)** LPS (T-independent stimulation) for 96 h. Production of different Ig isotypes (IgM, IgG1, IgG2a, IgG2b, IgG3) was measured by ELISA, using the appropriate supernatant dilutions; O.D. values obtained with supernatants from none-stimulated B cells were subtracted. Data show mean ± SD for four experiments (*n* = 8).

To explore the *in vivo* function of p38γ and p38δ in the antibody production, we immunised p38γ/δ^–/–^ and WT mice with either T-independent (TI) or T-dependent (TD) antigen. We monitored the humoral immune response to the TD antigen NP-KLH in alum and the TI antigen TNP-Ficoll measuring the levels of NP- or TNP-specific antibodies in the serum at different days post-immunisation by ELISA (Figure 5A and Supplementary Figure S5A). When mice were immunised with NP-KLH in alum, we found that p38γ/δ^–/–^ had a significantly lower IgG2 antibody response (IgG2a, 50% reduction; IgG2b, 25%) than WT mice. IgM, IgG1, IgG3 and IgA production were similar in both genotypes (Figure 5A). Analysis of the TI antibody response showed no major differences between the genotypes (Supplementary Figure S5A). These results suggest that p38γ and p38δ play a role in B cell antibody responses to T-dependent (TD), but not to T-independent (TI) antigens. The data also show that the reduction in the MZ B cell compartment in p38γ/δ^–/–^ mice has no major effect on antibody responses to T-independent antigens. Additionally, the ratio of high-affinity (NP_3_)/low-affinity (NP_30_) IgG2 antibodies was decreased in p38γ/δ^–/–^ compared to WT mice (Figure 5B), indicating that the production of high-affinity (NP_3_) IgG2a and IgG2b antibodies were reduced in p38γ/δ^–/–^ mice and suggesting that p38γ and p38δ are positive regulators of IgG2 antibody production to TD antigens *in vivo*.

We extended our analysis and, upon immunisation with a TD antigen, examined: GC B cells (CD95^+^GL7^+^), centrocytes and centroblasts; spleen and BM plasma cells (PC) (B220^low^ CD138^high^, IgM^+^ and IgG1^+^) and T follicular helper (Tfh) cells (CXCR5^+^PD-1^+^). We found that both p38γ/δ^–/–^ and WT mice generate similar frequency of GC B cells, PC and Tfh (Figures 5C–F). GC and PC analysis based on CD38 and PNA expression levels showed similar results (Supplementary Figure S6B).

To determine whether the specific IgG2a and IgG2b reduction in p38γ/δ^–/–^ mice resulted from an intrinsic B cell defect, we immunised CD19-Cre^KI/+^-p38γ/δ^f/f^ and p38γ/δ^f/f^ mice with NP-KLH in alum and found that the production of IgM, IgG1, IgG2a, IgG2b, IgG3 and IgA antibodies was similar between genotypes (Supplementary Figure S5B). Only a slight effect was detected for IgG2b production in CD19-Cre^KI/+^-p38γ/δ^f/f^ mice compare to controls; however, this was smaller than the those found in p38γ/δ^–/–^ mice (Supplementary Figure S5B). Additionally, we examined the ability of p38γ/δ^–/–^ B cells to class switch to IgG subclasses *in vitro*. In experiments using purified WT and p38γ/δ^–/–^ B cells with anti-CD40 plus IL-4 to mimic a TD response, we found no differences between these cells in class switch to the IgG isotypes analysed (Figure 5G). The data also suggest that the reduced *in vivo* antibody response to TD antigens is not B cell-autonomous. *In vitro* class switching experiments were also carried out using LPS to mimic a TI response and purified B cells; results were similar for WT and p38γ/δ^–/–^ B cells, with no detectable defects in class switch or antibody production due to lack of p38γ and p38δ (Figure 5H). The *in vitro* antibody production confirmed the *in vivo* results showing p38γ/δ^–/–^ B cell responses to T-independent antigen.

## Discussion

Here we study the effect of p38γ and p38δ on B cell development and function using mice lacking p38γ and/or p38δ in the whole animal, or mice deficient in p38γ and p38δ specifically in B cells. Analysis of BM showed that lack of p38γ and p38δ did not affect B cell differentiation in this compartment. In contrast, spleens from p38γ/δ^–/–^ mice, but not from CD19Cre^KI/+^-p38γ/δ^f/f^, were significantly smaller and cell numbers lower than in the spleen from other genotypes. p38γ and p38δ deficiency did not affect either the size or B cell number in lymph nodes, other secondary lymphoid organ (Risco et al., 2018). B cell ability to migrate *in vitro* in response to CXCL13 and CCL21 chemokines remained unaffected in absence of p38γ and p38δ, suggesting no B cell-intrinsic defects in homing and tissue compartmentalisation. The reduced B cell number in p38γ/δ^–/–^, but not in CD19Cre^KI/+^-p38γ/δ^f/f^, mouse spleen suggests a non-B cell-autonomous role for p38γ and p38δ in mature B cell development and maintenance. *In vitro* culture assays showed no marked differences in survival of p38γ/δ^–/–^ compared to WT B cells in response to BAFF. In addition, absolute numbers of the two mature B cell subsets, follicular and marginal zone, were also lower in p38γ/δ^–/–^ compared to single knockout and WT mice. An important role is attributed to B cells in MZ organisation and integrity (Nolte et al., 2003). We found that although MZ B cell frequency was affected in p38γ/δ^–/–^ mice, its organisation and architecture were unaltered in double knockout compared to WT mice. Additionally, MZ B cell frequency in CD19Cre^KI/+^-p38γ/δ^f/f^ mice was not affected compared to control p38γ/δ^f/f^ mice. These results indicate that the reduced frequency of MZ B cell in p38γ/δ^–/–^ mouse spleen is probably due to p38γ and p38δ function in other non-B-cell compartment. Since, marginal zone macrophages seem to play a role in B lymphocyte migration, adhesion and retention of B-cell in the MZ (Mebius et al., 2004), one hypothesis is that p38γ and p38δ regulate in MZ macrophages the expression of one or several molecules that are essential for B-cell migration, adhesion and/or retention. This hypothesis is supported by the fact that p38γ and p38δ control the production of cytokines and chemokines in macrophages in response to different agonists (Risco et al., 2012; Alsina-Beauchamp et al., 2018). Further studies using mice that lack p38γ and p38δ specifically in the myeloid compartment would help to elucidate the role of p38γ and p38δ in MZ B cells.

While immune synapse establishment was unaltered, p38γ and p38δ participate of the signalling cascade downstream the BCR. Expression of activation markers and costimulatory proteins was impaired in p38γ/δ^–/–^ B cells in response to low doses of anti-BCR. These experiments also identified a function for p38γ and p38δ in BCR-mediated immune responses, indicated by defective proliferation in response to anti-BCR, whereas lack of p38γ and p38δ had little effect on B cell proliferation in response to CD40 and no effect in response to LPS. These results coincide with those of previous studies showing that MEF2c, a general p38MAPK physiological substrate, regulates B cell proliferation in response to the BCR (Khiem et al., 2008; Wilker et al., 2008). Our data suggest that p38γ and p38δ participate in BCR-mediated signalling. MZ B cell differentiation requires weak BCR signals (Pillai and Cariappa, 2009); the reduction in B cell activation by low antigen doses in p38γ/δ^–/–^ mice might be responsible for the defect in MZ B cells.

p38γ and p38δ have a role in ERK1/2 activation in BM-derived dendritic cells and macrophages (Risco et al., 2012), but here we detected no apparent ERK1/2 activation defect in p38γ/δ^–/–^ B cells stimulated with anti-BCR. Contrary, CD40 activation of ERK1/2, which is mediated by the upstream MEK kinase, TPL2 (Eliopoulos et al., 2003), is abolished in p38γ/δ^–/–^ B-cells probably by the lack of TPL2 in these cells. The p38γ/p38δ-deficiency does not phenocopy the absence of TPL2 in B cells since TPL2^–/–^ B cell express normal levels of CD69 in response to anti-CD40 (Eliopoulos et al., 2003), whereas the expression of CD69 is impaired in p38γ/δ^–/–^ B cells in comparison with WT B cells, suggesting that p38γ/p38δ-signalling acts independently, at least in part, of TPL2.

The collective defects observed in p38γ/δ^–/–^ B cell activation and proliferation affected *in vivo* antibody responses, as p38γ/δ^–/–^ mice had significantly lower IgG2 levels than WT mice and showed defective class switching in response to immunisation with T-dependent antigen. Lack of both p38γ and p38δ nonetheless did not affect the ability of B cells to class switch to IgG *in vitro*, indicating that the reduced *in vivo* antibody response to T-dependent antigen is not B cell-autonomous and is probably due also to other intrinsic non-B-cell factors such as cell context. As shown for TLRs and Dectin-1 stimulation in p38γ/δ-deficient macrophages (Risco et al., 2012; Alsina-Beauchamp et al., 2018), impaired cytokine production might be involved in the defective T-dependent antibody response. In addition, we have previously shown evidence of the role of p38γ/p38δ in the regulation of B cell responses to collagen, since IgG2 levels were significantly lower in p38γ/δ^–/–^ than in WT mice in a collagen induced arthritis model (Criado et al., 2014). Also, we showed that p38γ/δ^–/–^ T cells produce less IFNγ in response to α-CD3 when compare to WT control T cells (Criado et al., 2014). Since T cell-derived IFNγ is implicated in IgG2a class switching (Peng et al., 2002), our data indicate that loss of p38γ and p38δ affects the ability to switch from IgM to IgG2a and IgG2b *in vivo* in response to TD antigens, possibly by reducing IFNγ secretion of T cells. Since humoral adaptive immunity is regulated by CD40 engagement, we cannot rule out the possibility that defect in CD40 signalling observed in p38γ/δ^–/–^ B cells can cause a decrease in the *in vivo* TD-IgG2 production.

## Conclusion

In this work, we unveil an important role of the alternative p38MAPK, p38γ and p38δ, in B cell development and response, which is opposed to what has been described for the canonical p38MAPK, p38α. Experiments performed using p38α^–/–^ chimeric mice show that this kinase is not essential for either B cell development, LPS-, CD40- or BCR-driven proliferation of mature B cells, or for the production of normal serum Ig levels (Kim et al., 2005). Our data indicate that p38γ and p38δ are important components of the BCR and CD40 signalling pathways, necessary for robust B cell development and expansion, and thus for the *in vivo* antibody response. Our results show the importance of p38γ and p38δ in B cell proliferation, whose dysregulation can lead to an inadequate immune response, to immunodeficiency, autoimmune disease and even to leukemia, and suggest that impairment or lack of p38γ and p38δ function leads to B cell-related disorders.

## Data Availability Statement

The datasets generated for this study are available on request to the corresponding authors.

## Ethics Statement

The animal study was reviewed and approved by CSIC Committee on Ethics and Animal Welfare and the Comunidad de Madrid (References PROEX 316/15 and PROEX 071/19).

## Author Contributions

LB, SR-G, ED-M, AR, RJ-S, YC, and AC designed and performed the experiments, analysed the data. YC and AC wrote the manuscript.

## Conflict of Interest

The authors declare that the research was conducted in the absence of any commercial or financial relationships that could be construed as a potential conflict of interest.

## Abbreviations

FO: follicular
GC: germinal centre
MZ: marginal zone
PC: plasma cells
RT: room temperature
su-Ag: surrogate antigen.
