# Supplementary material for: B Cell Development and T-Dependent Antibody Response Are Regulated by p38γ and p38δ
Source: Front Cell Dev Biol. 2020 Mar 24;8:189. doi: 10.3389/fcell.2020.00189 (PMC7105866; doi:10.3389/fcell.2020.00189)
Supplement: Supplementary file 1 [file Data_Sheet_1.PDF]

Supplementary Figure legends

Figure S1. p38MAPK expression in B cells isolated from the spleen of WT, p38$\gamma/\delta^{-/-}$, p38$\gamma/\delta^{f/f}$ and CD19-Cre$^{KI/+}$-p38$\gamma/\delta^{f/f}$ mice. (**A**) Lysates from B cells were immunoblotted with anti-total p38α, -p38γ and -p38δ antibodies. Representative western blots are shown. (**B**) Tissue lysates from colon, pancreas and lung were immunoblotted as in (A). Representative western blots are shown. Anti-p38γ and anti-p38δ are polyclonal antibodies raised in sheep against recombinant protein as described in (6). Anti-p38δ (A, B) and anti-p38γ (A, B) recognise an unspecified band (red asterisk). p38δ and p38γ protein levels in B cells from p38$\gamma/\delta^{f/f}$ are noticeably lower than that in WT B cells (A).

Figure S2. Analysis of B cell populations in the spleen. (**A**) Total cell number in spleens of adult mice of the indicated genotypes. Each dot represents a single mouse (n= 6-12). (**B**) Representative photographs of spleens from adult WT and p38$\gamma/\delta^{-/-}$ mice. (**C**) Total cell number in spleens of adult mice of the indicated genotypes. Each dot represents a single mouse (n= 6-10). (**D**) B cell frequency and (**E**) total number in spleens of adult mice of the indicated genotypes; B cells were gated as CD19^{+} cells. Each dot represents a single mouse (n= 8-10). (**F**) Splenocytes from adult WT and p38$\gamma/\delta^{-/-}$ mice were stained with anti-CD3, -CD4, -CD8, -CD11b, -F4/80 and –Gr1; the total cell number of the indicated populations was determined after flow cytometry analysis. Each dot represents a single mouse (n= 14-18). (**G**) Representative dot plots for CD21 and CD23 expression in splenocytes from mice of the specified genotypes. Frequencies of gated follicular (FO) B cells (CD21^{+}CD23^{+}) and marginal zone (MZ) B cells (CD21^{hi}CD23^{-}) are indicated. (**H**) Frequency and total cell number of FO and MZ B cells in adult mouse spleens. Each dot represents a single mouse (n= 6). No statistically significant differences were found. ns, not significant, * $p < 0.05$, ** $p \leq 0.01$.

Figure S3. Effect of p38γ or p38δ on B cell activation (**A**-**C**) Splenocytes from WT, p38$\gamma^{-/-}$ or p38$\delta^{-/-}$ mice were cultured alone (none) or with anti-BCR (1 and 10 μg/ml) for 20 h. $CD19^+$-gated B cells were analysed for expression of CD69 and CD86 activation markers by flow cytometry. (**A**) Representative profiles of surface CD69 expression (filled histograms) in B cells stimulated with the indicated ligands; grey line, isotype control. (**B**) Frequency of activated B cells, gated as $CD69^+CD86^+$, in the total B cell population for each condition. (**C**) CD69 mean fluorescence intensity (MFI) levels in $CD19^+$-gated B cells. In A and C each dot represents a single mouse (n = 6). (**D**) Violet tracer-labelled splenocytes of the specified mouse genotypes were cultured alone (none) or with anti-BCR (1 and 10 μg/ml) plus IL-4 (20 ng/ml) for 96 h. $CD19^+$-gated B cells were analysed for violet tracer signal dilution by flow cytometry. Representative violet tracer profiles of WT (filled histograms) and p38 isoform-deficient (black line) B cells stimulated as above; grey line, violet tracer profile of unstimulated B cells after 96 h in culture (left panel). Proliferation index of B cells in each condition; values obtained with FlowJo software. Each dot represents a single mouse (*n* = 4-5) (right panel). (**E**-**F**) Splenocytes from mouse of the specified genotypes were cultured alone (none) or with the TLR4 ligand LPS (2.5 μg/ml) for 20 h. $CD19^+$-gated B cells were analysed for expression of CD69 and CD86 by flow cytometry. (**E**) Representative profiles of CD69 expression (filled histograms) in B cells stimulated with the indicated ligands; grey line, isotype control. (**F**) Frequency of activated B cells (upper panel), gated as $CD69^+CD86^+$, and CD69 mean fluorescence intensity (MFI) levels (bottom panel) in the total B cell population for each condition. Each dot represents a single mouse (n = 6). (**G**) Violet tracer-labelled splenocytes of the specified mouse genotypes were cultured alone (none) or with LPS (2.5 μg/ml) for 96 h. $CD19^+$-gated B cells were analysed for violet tracer signal dilution by flow cytometry. (Left panel) Representative violet tracer profiles of WT (filled histograms) and p38 isoform-deficient (black line) B cells stimulated as above; grey line, violet tracer profile of unstimulated B cells after 96 h in culture. (Right panel) Proliferation

index of B cells in each condition; values obtained with FlowJo software. Each dot represents a single mouse (n = 4-5). ns, not significant; * $p < 0.05$; ** $p \leq 0.01$; *** $p \leq 0.001$; p value is also indicated in number.

Figure S4. Effect of p38γδ deficiency in MZ and FO B cell activation. Total splenocytes from WT and p38γδ deficient mice were CD21/CD23 stained and MZ ($CD21^{high}$ $CD23^{low}$) and FO ($CD21^{+}$ $CD23^{+}$) B cell populations sorted. (**A**) Representative dot plots of CD21/CD23 of the pre-sorted and post-sorted populations are shown. (**B-C**) Sorted MZ and FO B cells were cultured alone or with anti-BCR (1 and 10 μg/ml), anti-CD40 (1 μg/ml) or LPS (2.5 μg/ml) for 24 h, and then analysed for surface expression of CD69, CD86 and CD25 by flow cytometry. Frequency of $CD69^{+}$, $CD86^{+}$ and $CD25^{+}$ B cells (upper panels), and mean fluorescence intensity (MFI) values for each marker (bottom panels) in the MZ (**B**) and FO (**C**) B cell populations for each condition. Each dot represents an experiment with sorted populations from a pool of 2 spleens (n=6 mice for WT; n=8 mice for p38γδ deficient). * $p < 0.05$; *** $p \leq 0.001$; p value is also indicated in number.

Figure S5. Antibody response in p38γ- and p38δ-deficient mice. (**A**) Adult WT and $p38\gamma/\delta^{-/-}$ mice were immunized i.p. with TNP-Ficoll (T-independent antigen). Serum samples were analysed by ELISA for TNP-specific antibodies of different isotypes (IgM, IgG1, IgG2a, IgG2b, IgG3) at the indicated times. WT (black circles) and $p38\gamma/\delta^{-/-}$ mice (white circles). O.D., optical density. (**B**) Adult $p38\gamma/\delta^{f/f}$ and $CD19\text{-}Cre^{KI/+}\text{-}p38\gamma/\delta^{f/f}$ mice were immunized i.p. with NP-KLH in alum (T-dependent). Serum samples were analysed by ELISA for TNP-specific antibodies of different isotypes (IgM, IgG1, IgG2a, IgG2b, IgG3, IgA) at the indicated times. O.D., optical density. Each dot in A and B, represents a mouse (n = 4 or 5 mice/group).

Figure S6. Analysis of GC B cells and PC upon T-dependent immunization. Adult WT and p38$\gamma/\delta^{-/-}$ mice (*n*=5 mice per group) were immunized i.p. with NP-KLH in Alum, and analysed at day 7 post-immunization for GC B cells and PC populations in spleen and bone marrow by flow cytometry. (**A**) Gating strategy for GC B cell population based on B220/IgD/CD95/GL7 expression in total splenocytes. (**B**) Gating strategy for GC B cells (PNA^{high} CD38^{low}) and PC (CD38^{high} B220$^{low/neg}$) based on B220/IgD/PNA/CD38 expression in total splenocytes. Frequency of GC B cells and PC are shown; each dot is a mouse. (**C**) Gating strategy for PC populations based on B220/CD138/IgG1/IgM expression in total splenocytes and bone marrow.

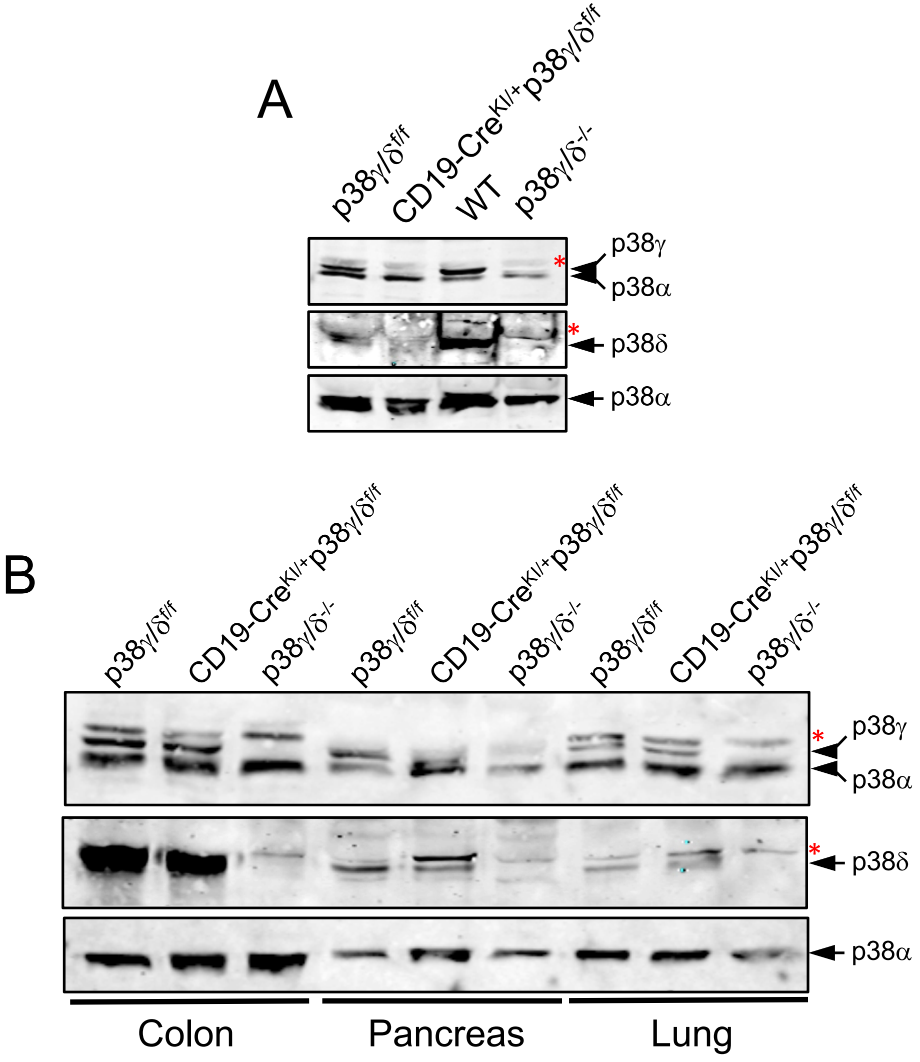

Barrio et al. Fig. S1

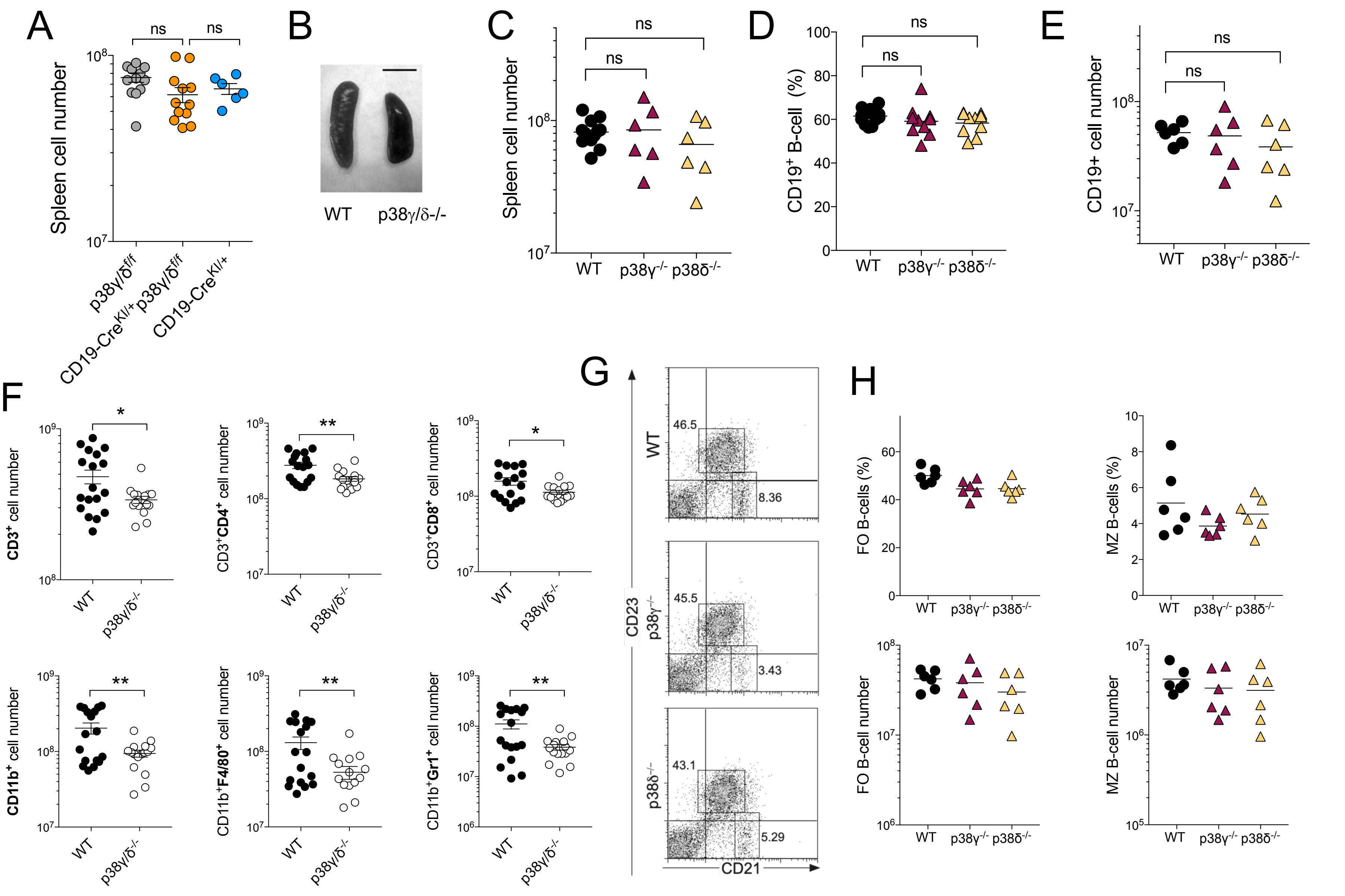

Barrio et al. Fig. S2

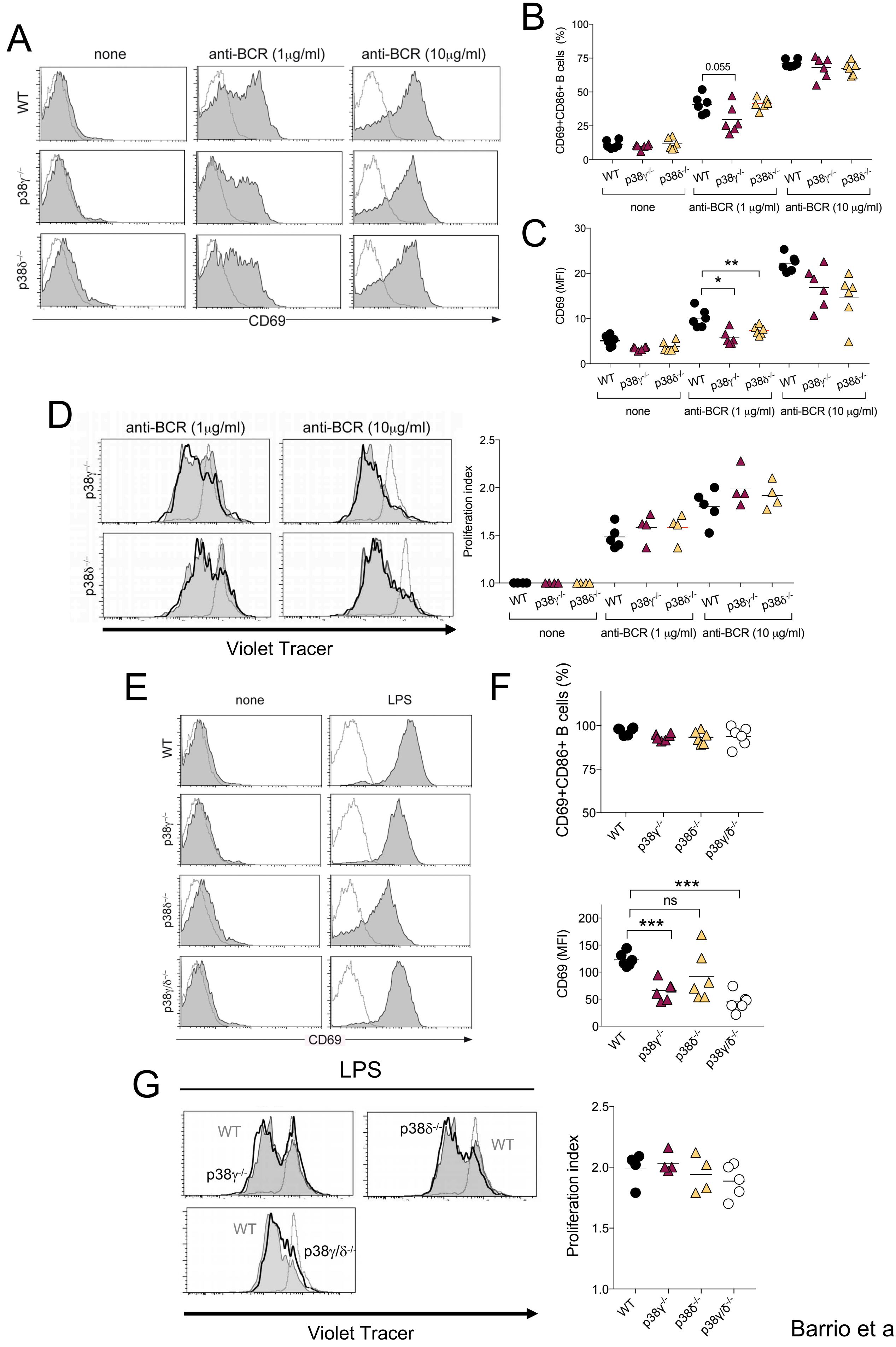

Barrio et al Fig. S3

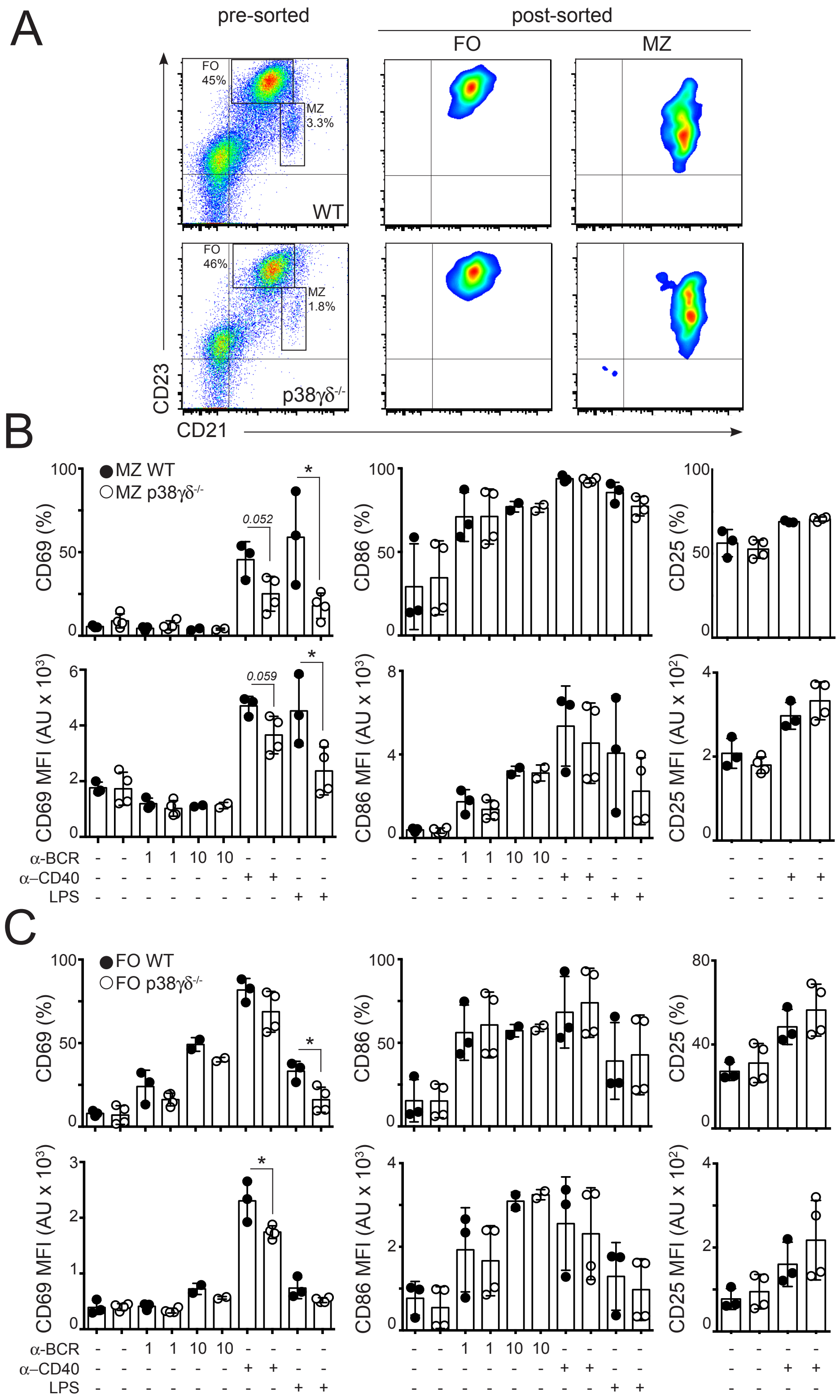

Barrio et al Fig S4

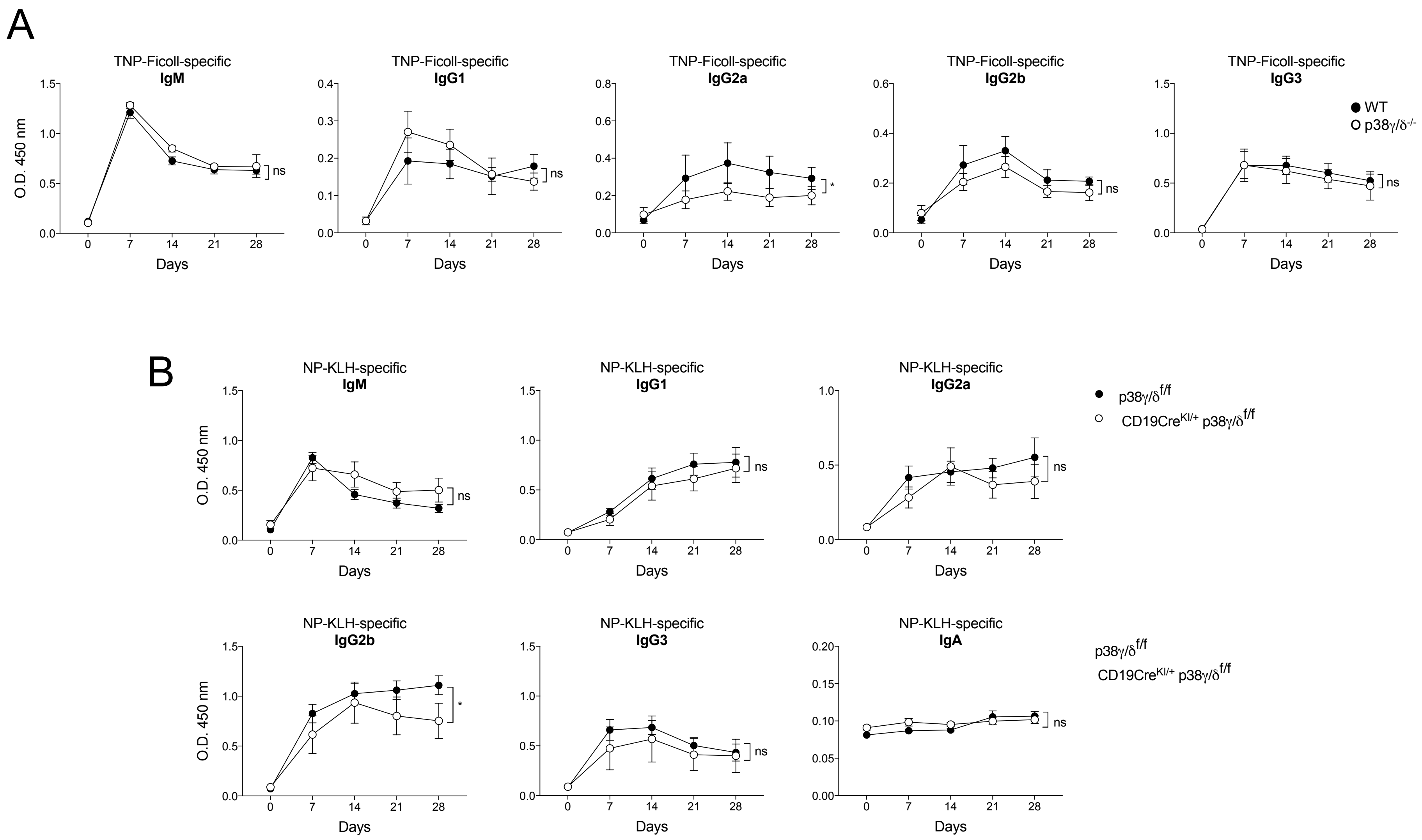

Barrio et al Fig S5

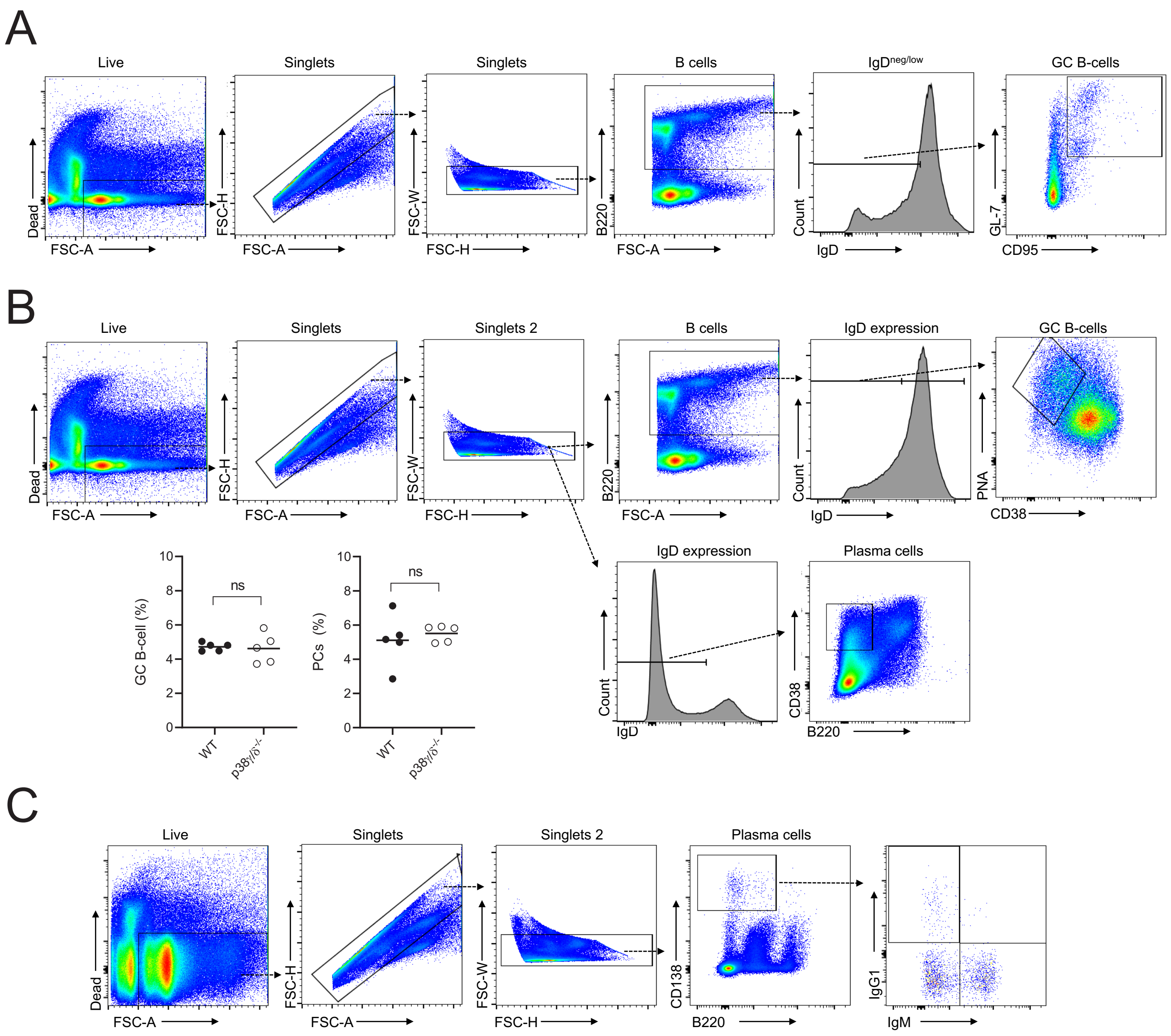

Barrio et al Fig S6
